# Consequences of PPAR_****α****_ Invalidation on Glutathione Synthesis: Interactions with Dietary Fatty Acids

**DOI:** 10.1155/2011/256186

**Published:** 2011-09-12

**Authors:** Najoua Guelzim, Jean-François Huneau, Véronique Mathé, Annie Quignard-Boulangé, Pascal G. Martin, Daniel Tomé, Dominique Hermier

**Affiliations:** ^1^INRA, UMR914 Nutrition Physiology and Ingestive Behavior, F-75005 Paris, France; ^2^AgroParisTech, UMR914 Nutrition Physiology and Ingestive Behavior, F-75005 Paris, France; ^3^INRA, UR66 ToxAlim, Laboratoire de Pharmacologie et Toxicologie, Toulouse, France

## Abstract

Glutathione (GSH) derives from cysteine and plays a key role in redox status. GSH synthesis is determined mainly by cysteine availability and **γ**-glutamate cysteine ligase (**γ**GCL) activity. Because PPAR**α** activation is known to control the metabolism of certain amino acids, GSH synthesis from cysteine and related metabolisms were explored in wild-type (WT) and PPAR**α**-null (KO) mice, fed diets containing either saturated (COCO diet) or 18 : 3 n-3, LIN diet. In mice fed the COCO diet, but not in those fed the LIN diet, PPAR**α** deficiency enhanced hepatic GSH content and **γ**GCL activity, superoxide dismutase 2 mRNA levels, and plasma uric acid concentration, suggesting an oxidative stress. In addition, in WT mice, the LIN diet increased the hepatic GSH pool, without effect on **γ**GCL activity, or change in target gene expression, which rules out a direct effect of PPAR**α**. This suggests that dietary 18 : 3 n-3 may regulate GSH metabolism and thus mitigate the deleterious effects of PPAR**α** deficiency on redox status, without direct PPAR**α** activation.

## 1. Introduction

PPAR*α* is a major regulator of the macronutrient metabolism, especially during the fed-to-fasting transition [1]. Formerly, PPAR*α* has been involved in the regulation of lipid metabolism, including cellular uptake of fatty acids, intracellular fatty acid binding and activation, microsomal *ω*-oxidation, *β*-oxidation and ketogenesis, and synthesis of lipoproteins [2, 3]. Later on, PPAR*α* effects have been shown to extend to a number of target genes involved in the metabolism of glucose, glycerol and glycogen, and bile acids, as well as in inflammation, detoxification, and hepatocarcinogenesis [4, 5]. More recently, PPAR*α* has also been shown to play a role in amino acid metabolism, through the regulation of a number of hepatic target genes involved in transamination, deamination, and urea synthesis [6–8]. 

Beyond nutritional situations, interest in PPAR*α* effects on amino acids metabolism can also be considered in light of the involvement of specific amino acids in physiopathological processes associated with the metabolic syndrome. We have recently shown that PPAR*α* deficiency decreases whole body nitric oxide (NO) synthesis from arginine, suggesting a beneficial effect of PPAR*α* on vascular function [9]. Cysteine is a second amino acid of which metabolism might be of importance in the context of metabolic syndrome. Indeed, cysteine is the rate-limiting substrate for the synthesis of glutathione (GSH) [10], a major endogenous antioxidant, protecting cells from reactive oxygen species (ROS). Most of the GSH is utilized in antioxidant defence via the glutathione peroxidase (GPX) enzyme family to neutralize ROS and protect the body from their noxious effect [11]. GSH synthesis is a two-step process. The first rate-limiting step is the condensation of cysteine and glutamate to *γ*-glutamylcysteine and is catalyzed by *γ*-glutamate cysteine ligase (*γ*GCL). While GSH synthesis occurs in every tissue, the liver plays a prominent role in whole body GSH flux [12]. A growing number of studies support a link between glutathione synthesis and utilization and the metabolic syndrome. Alterations in glutathione status and utilisation are long-recognized hallmarks of metabolic syndrome-associated oxidative stress [13–17]. In parallel, fuelling glutathione synthesis through an extra cysteine supply has been shown to alleviate insulin resistance and oxidative stress in animal models of the metabolic syndrome [18, 19]. 

Despite that PPAR*α* activation enhances ROS generation by activating fatty acid and *β*- and *ω*-oxidation, it can also promote ROS clearance through increased expression and/or activity of antioxidant enzymes, such as catalase (CAT) and superoxide dismutase (SOD) [20–23]. Accordingly, PPAR*α* deficiency decreases the activity of the same enzymes [23]. However, little is known regarding the role of PPAR*α* in hepatic metabolism of GSH. In fasted mice, PPAR*α* deficiency reduced hepatic GSH level and GPX activity [23]. Consistently, fibrate treatment increased erythrocyte GPX activity in human [24] and hepatic GSH content in the mouse [25]. A few studies have also addressed the effect of n-3 polyunsaturated fatty acids (PUFA), natural PPAR*α* ligands, on GSH metabolism. In a rat model of chronic heart failure, n-3 fatty acid treatment increased cardiac **γ**GCL content and activity, increased total and reduced glutathione, and decreased oxidized glutathione [26]. As concerns the destruction of ROS, the effects of n-3 PUFA supplementation in the rat are inconsistent, with GPX activity being decreased [27] or not changed [28]. In cultured human fibroblasts, DHA (22 : 6 n-3) induced expression and activity of **γ**GCL, as well as intracellular GSH content [29]. However, none of these studies provided a direct evidence for a role of PPAR*α* in the modulation of GSH metabolism by n-3 fatty acids. 

The first and main objective of the present study was to investigate the effects of PPAR*α* invalidation in the regulation of GSH metabolism by using wild-type (WT) and PPAR*α*-deficient mice (KO). We have shown previously in the mouse that a number of hepatic genes known to be regulated essentially via PPAR*α* were upregulated by *α*-linolenic acid (ALA, 18 : 3 n-3) as they are by its long-chain derivatives. This was observed in WT mice fed rather high-fat diets [30] but also in WT mice fed low-fat diets, albeit to a lesser extent [31]. For these reasons, as a secondary objective, we aimed to assess the contribution of dietary n-3 PUFA to PPAR*α* activation, by exposing mice to contrasted diets, containing mostly either saturated FA or ALA. We explored GSH synthesis from cysteine, as well as hepatic thiol content and mRNA levels of enzymes involved in the protection against oxidative damages.

## 2. Materials and Methods

### 2.1. Animals and Diets

Male PPAR*α*-deficient mice [32] were supplied by the ToxAlim laboratory (UR66, INRA, Toulouse), in which several additional rounds of backcrossing have been performed initially to increase the C57BL/6J genetic background and to generate the animals used [33]. Wild type male C57BL/6J mice were obtained from Charles River (L'Arbresle, France). *In vivo* studies were conducted under European Union guidelines for the use and care of laboratory animals. 

Twenty-eight 6-7-week-old mice were bred in INRA's facility in Paris and housed collectively on wood litter, at 22 ± 2°C under 12-h light/dark cycles (light on at 06:00 am). They were fed *ad libitum* a standard pelleted diet (Teklad 20-18S, Harlan, Gannat, France) and acclimated to local conditions for 4 weeks. At 10-11 weeks of age, mice were fed during 8 weeks one of the two experimental diets differing in their fatty acid profile (LIN or COCO diet, as described below). They had free access to food and tap water. Food consumption (as assessed per collective cage and expressed relatively to the mean body weight of mice in each cage) and individual body weight were recorded weekly.

Diets were provided as pellets by UPAE-INRA (Jouy-en-Josas, France) as described previously [31]. The calculated composition (in weight) of the two diets was 21.0% protein, 69.2% carbohydrate, 4.8% lipid, 4.0% vitamins, and 4.0% minerals. The experimental diets were isoenergetic, with lipids providing 11.3% of total energy intake. The choice of a low fat diet was based on the results of a previous nutrigenomic study of some of the present authors, showing significant effects of PPAR*α* deficiency on lipid and xenobiotic metabolism in mice fed the same diets as in the present study [31]. Besides, in our previous studies, Cyp4a14 gene, exhibiting a PPRE sequence and being specifically activated by PPAR*α* pure agonists [34], was significantly more expressed in WT mice than in KO mice fed a low fat diet rich in 18 : 3 n-3 [9, 31]. This indicated that even a low dietary amount of n-3 PUFA was able to activate PPAR*α*, which justified the choice of the dietary conditions. Oils used for experimental diet preparation were hydrogenated coconut oil for a saturated FA-rich diet (SFA, COCO diet) and linseed oil for a ALA-rich oil (LIN diet). Their fatty acid composition was (in weight %): 99.6% SFA, 0.3% monounsaturated FA (MUFA), and 0.1% PUFA for the COCO diet; 9.0% SFA, 18.0% MUFA, and 72.9% PUFA (58% ALA) for the LIN diet [31].

### 2.2. Experimental Design

At the end of the experimental period, mice were fasted for 5 h, then weighed and anesthetized with combined xylazine/ketamine solution. Blood was taken by cardiac puncture, and mice were then killed by exsanguination. Plasma was separated by centrifugation (1,700∗g, 20 min, 4°C) and aliquots were stored at −80°C. The abdominal cavity was then opened, and the liver was removed and weighed. Several liver samples were snap frozen in liquid nitrogen and stored at −80°C. Epididymal adipose tissue (EpAT, visceral localization) and inguinal adipose tissue (IngAT, subcutaneous localization) were removed and weighed.

### 2.3. Biochemical Analyses

Blood glucose concentration was determined with an Accu-Chek glucometer (Roche Diagnostics, Meylan, France). Plasma cholesterol, triglycerides, and uric acid were determined by colorimetric enzymatic methods using commercial kits (Bio-Merieux, Craponne, France), adapted for use in a 96-well microplate reader (Molecular Devices, Saint-Grégoire, France). Plasma amino acids were determined by ion-exchange chromatography with postcolumn ninhydrine derivatisation on an Aminotac JLC-500/V (Jeol, Tokyo, Japan). Plasma concentrations of adiponectin, plasminogen activator inhibitor-1 (PAI-1), monocyte chemotactic protein-1 (MCP-1), leptin, and insulin were determined using multiplexed immunoassays (Millipore-Linco Research, St. Charles, USA) on a Bioplex-200 analyzer (Bio-Rad Laboratories, Marnes-la-Coquette, France).

### 2.4. Gene Expression

Total RNA was extracted from a liver sample using Trizol reagent (Invitrogen, Carlsbad, USA), and synthesis of cDNA was performed on 400 ng of total RNA using a high capacity cDNA reverse transcription kit, based on the use of both oligodT and hexamers (Applied Biosystems, Foster City, USA). The primers listed in Table 1 were used for quantitative PCR on a 7300 real-time PCR system (Applied Biosystems), as described previously [35]. Gene expression was determined using the 2^−ΔCt^ formula where ΔCt = (Ct  target  gene − Ct  18S).

### 2.5. Hepatic Thiol Concentrations

Total low-molecular weight thiols (cysteine, GSH, and cysteinylglycine (CysGly)) were analyzed using high-performance liquid chromatography (HPLC) as described previously, with slight modifications [36]. Briefly, 50 mg of liver samples were homogenized in 950 *μ*L of 0.1 M phosphate buffer and centrifuged (3,000∗g, 5 min). A small volume of supernatant was removed for subsequent protein assay with the Pierce bicinchoninic acid (BCA) Assay Kit (Pierce, Rockford, USA). Sixty *μ*L of the supernatant were reduced with 0.66 vol of triphenylphosphine (10% in dimethylformamide) deproteinized by 2 vol of 10% TCA and derivatized by 3% 4-(aminosulfonyl)-7-fluoro-2,1,3-benzoxadiazole at pH 9.0. After 1 hour incubation at 4°C, derivatization was stopped by adding 25 *μ*L of 4 mM HCL. N-acetylcysteine was added in every sample as an internal standard. Thiol separation was achieved at 45°C on a Kromasil c18 column (15 cm × 4.6 mm id., 3.5 *μ*m) with a mobile phase consisting in 90% 100 mM citrate buffer pH 4.0 and 10% methanol. External standards of GSH, cysteine, and CysGly were used for the identification and quantification of thiols in liver homogenates. Results were normalized for liver weight or protein content.

### 2.6. Hepatic *γ*GCL Activity

Liver *γ*GCL activity was assessed in liver homogenate using a fluorescence-based method as described previously [37]. Briefly, a cytosolic fraction was prepared from a 50 mg liver homogenate by successive centrifugation (10,000∗g, 10 min, 4°C and 15,000∗g, 5 min, 4°C). Fifty *μ*L of this cytosolic fraction were preincubated for 5 min at 37°C with 1 vol of reaction medium consisting in (final concentration): 133 mM Tris, 13.3 mM ATP, 6.66 mM glutamic acid, 0.66 mM serine, 0.66 mM EDTA, 6.66 mM sodium borate and 13.3 mM MgCl_2_. The reaction was started by the addition of 50 *μ*L of cysteine (0.66 mM, final concentration) and stopped after 20 minutes at 37°C with 50 *μ*L of 200 mM sulfosalicylic acid followed by centrifugation at 2,000 ×g. For derivatization, 20 *μ*L of the resulting supernatant were incubated with 180 *μ*L of 10 mM 2,3-naphthalenedicarboxaldehyde (NDA) solution, to form NDA-*γ*-glutamylcysteine. Fluorescence intensity (*ε*_ex_472 nm − *ε*_em_528 nm) was measured on a fluorescence plate reader (CytoFluor 4000, Applied Biosystems) and quantified using standard curves of NDA-GSH. Results were corrected for initial GSH content and normalized for liver weight or protein concentration of the cytosolic fraction.

### 2.7. Statistical Analyses

Data are presented as means ± SEM. They were analysed using the SAS program (SAS Institute, Cary, USA). Differences between treatments and interactions were tested with a two-way ANOVA with genotype and diet as factors, using the GLM procedure. When the genotype and/or diet factor was significant, differences between means were tested for significance using the *post hoc *Tukey-Kramer procedure. Significance level was set at *P* < 0.05.

## 3. Results

### 3.1. Markers of PPAR*α* Deficiency in Relation to Dietary Treatment (Table 2)

Body weight was higher in the KO mice. Throughout the 8 weeks of the experiment, individual daily food intake (in g, and as estimated from total food consumed per cage) was higher in KO mice, but was similar to the WT mice when corrected for the body weight (130 ± 14 mg/g body weight per day, whatever the genotype or the diet). This indicated that the higher body weight of the KO groups did not differ primarily from a higher food intake. In WT mice, the fatty acid composition of the diets did not affect the markers of the metabolic syndrome. PPAR*α* deficiency resulted in higher body and liver weights, as well as in adipose tissue proportion than in WT mice. *Post-hoc* analysis showed that this genotype effect was significant only in the COCO-fed mice, and not in the LIN-fed ones. In contrast, there was no overall effect of PPAR*α* deficiency on liver proportion but a significant genotype ∗ diet interaction, so that *post-hoc* analyses revealed that the liver proportion was significantly increased by PPAR*α* deficiency the COCO-fed group, and not in the LIN-fed one. Similarly, the higher plasma concentrations of triglyceride and cholesterol found in the KO mice were more pronounced in the COCO-fed group than in the LIN-fed one. In contrast, when compared to their WT counterparts, KO mice exhibited a lower glycemia, this effect being more pronounced in mice fed the LIN diet.

### 3.2. GSH Metabolism Related Parameters

#### 3.2.1. Hepatic Thiols Status (Table 3)

Hepatic GSH concentration and pool varied according to the experimental conditions, with a strong interaction between the genotype and the diet (*P* < 0.001). In WT mice, GSH concentration and pool were 40% higher in those fed the LIN diet than in those fed the COCO diet. PPAR*α* deficiency resulted in increased concentration and pool of GSH in the COCO-fed mice, while it did not affect the LIN-fed diet ones. Hepatic concentration and pool of cysteine and CysGly were much lower than those of GSH and were neither affected by PPAR*α* deficiency nor by the diet.

#### 3.2.2. Hepatic *γ*GCL Activity (Figure 1) and mRNA Levels of *γ*GCL and CDO (Table 4)

**Figure 1:**
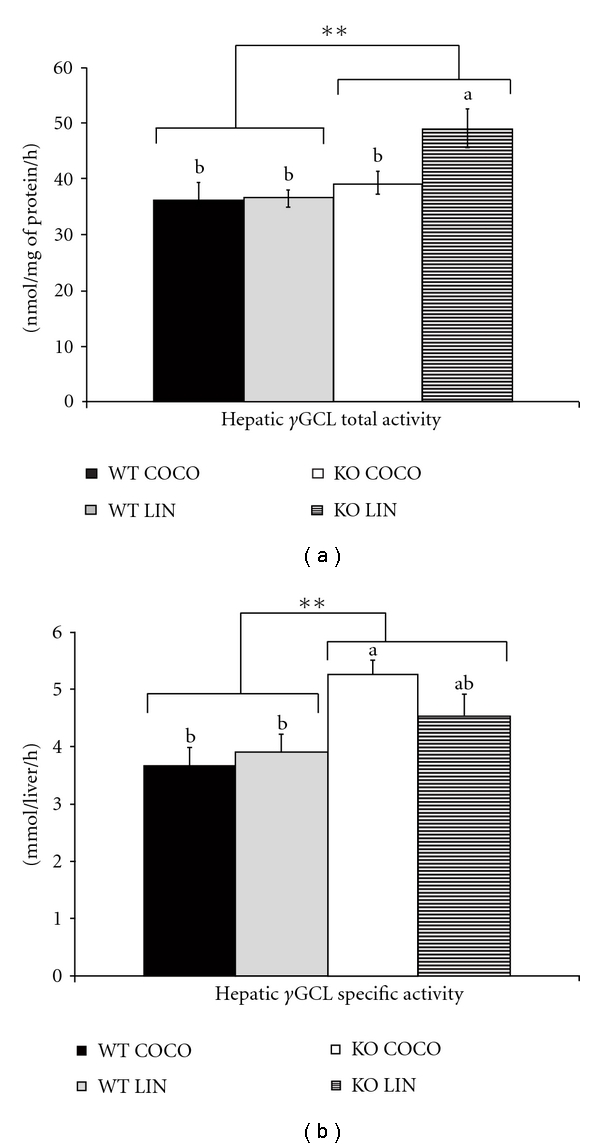
Hepatic GCL activity in WT and PPAR*α*-deficient (KO) mice fed diets containing either saturated FA (COCO diet) or ALA (LIN diet) for 8 weeks. Values are expressed as nmol/mg of protein/h for specific activity (a) and as mmol/liver/h for total activity (b). They are means ± standard errors for 7 mice per group, **KO group significantly different from WT group *P* < 0.01. Columns sharing a same superscript letter, or without superscript letter, were not significantly different at *P* < 0.05.

In WT mice, the fatty acid composition of the diets did not influence either specific or total ***γ***GCL activity. PPAR*α* deficiency significantly increased ***γ***GCL specific activity (Figure 1(a)) and total activity (Figure 1(b)). The *post-hoc* analysis showed that the effect of PPAR*α* deficiency on ***γ***GCL was significant only in the LIN-fed mice for the specific activity and in the COCO-fed mice for the total activity. Hepatic mRNA level of ***γ***GCL and CDO was not affected by either PPAR*α* deficiency or the diet (Table 4).

#### 3.2.3. Plasma Amino Acid Concentrations (Table 5)

Among amino acids related to cysteine metabolism, PPAR*α* deficiency was associated with a significantly higher plasma concentration of methionine and lower concentration of glycine and taurine. Plasma concentration of glutamic acid and cysteine was not affected by the genotype. None of the plasma concentrations was influenced by the diet.

### 3.3. Oxidative Stress and Inflammatory Status

#### 3.3.1. Hepatic mRNA Levels of Antioxidant Enzymes and of Inflammatory Markers (Table 4)

Hepatic mRNA levels of the genes coding for GPx (Gpx1), CAT (Cat), and Cu/ZnSOD (Sod1) were not affected by PPAR*α* deficiency, while PPAR*α* deficiency significantly increased the mRNA level coding for MnSOD (Sod2). None of these mRNA levels was influenced by the diet, whatever the genotype.

 CD68 mRNA level was significantly higher in KO mice than in WT ones. *Post-hoc *analysis showed that this genotype effect was borne by the mice fed the LIN diet essentially. SAA and MCP1 mRNA levels were also numerically higher in KO mice, but the difference was not statistically significant (*P* = 0.0684 for SAA and 0.0829 for MCP1).

#### 3.3.2. Plasma Concentration of Uric Acid (Figure 2)

In WT mice, the fatty acid composition of the diets did not influence uric acid concentration. When compared to their WT counterparts, PPAR*α* KO mice exhibited a higher plasma uric acid concentration. However, due to a significant genotype*diet interaction, this concentration was affected only in mice fed the COCO diet.

#### 3.3.3. Plasma Cytokines and Hormones (Table 6)

Concentrations of leptin, insulin, and PAI1 were neither affected by the diet nor by the genotype. Independently of the genotype, adiponectine concentration was significantly higher in the LIN-fed mice diet than in the COCO-fed mice and tended to decrease (*P* = 0.0596) in response to PPAR*α* deficiency. In contrast, MCP1 concentration was not affected by the diet, but significantly decreased in PPAR*α*-deficient mice compared to WT mice.

## 4. Discussion

PPAR*α* KO has been previously shown to affect fatty acid metabolism [33] and glucose homeostasis [1, 38]. In line with these observations, our experimental conditions reproduced the characteristic phenotypic alterations associated with PPAR*α* deficiency, which are similar to some of those clustered in the metabolic syndrome, such as obesity, hepatic hypertrophy, hypertriglyceridemia, hypercholesterolemia, and glycemic dysregulation (Table 2). Because alteration of glutathione metabolism is a common feature of the metabolic syndrome [39], and since we and others have previously shown that PPAR*α* invalidation impacted some specific amino acid metabolic pathways [6–9, 40], investigating the effects of PPAR*α* deficiency on cysteine metabolism and GSH status was especially relevant. 

### 4.1. Consequences of PPAR*α* Deficiency on GSH Metabolism

Under our experimental conditions, PPAR*α* invalidation was primarily associated with an overall increase in the hepatic pool of GSH (*P* < 0.0367, Table 3). The significant genotype ∗ diet interaction (*P* < 0.001) showed that this was true only in mice fed the COCO diet, as discussed below. An increase in GSH pool might reflect an increase in GSH synthesis and/or a decrease in GSH utilization (export and/or degradation). As concerns GSH synthesis, it is regulated primarily by *γ*GCL activity, cysteine availability, and GSH feedback inhibition [41]. In PPAR*α*-deficient mice, the increase in hepatic GSH pool could be directly related to an enhanced synthesis from cysteine, as suggested by their higher total *γ*GCL activity (Figure 1(b)). In parallel, PPAR*α* invalidation was accompanied by a lower plasma concentration of one of the GSH precursors, glycine, but also of taurine, whereas that of methionine increased (Table 5). In contrast, plasma concentration of cysteine was not affected, suggesting that cysteine availability was not limiting for GSH synthesis. Our results on taurine are consistent with the decrease in plasma taurine concentration and in CDO mRNA level in the adipose tissue of obese mice [42]. The blunting of cysteine to taurine flux in the adipose tissue may eventually result in a sparing of cysteine which could be used for glutathione synthesis. Because part of glycine synthesis involves methyl transfer from methionine, the decrease in glycine, together with the increase in methionine, may reflect alterations in one-carbon metabolism, as reported in subjects with nonalcoholic hepatosteatitis [43]. 

In addition to an increase in synthesis rate, the higher hepatic GSH pool in KO mice could also result from a decreased utilization in antioxidant defences. Under our experimental conditions, the level of GPX1 mRNA, the major glutathione peroxidase isoform, was unaffected by PPAR*α* deficiency (Table 4). This is consistent with a previous study showing that GPX activity was not altered in fasted PPAR*α* KO mice [23] and suggests that fibrate-enhanced GPX activity found in human erythrocytes [24] was PPAR*α* independent, and probably involved complex post-transcriptional regulations.

### 4.2. Interactions between Genotype and Diet

A secondary aim of this study was to investigate the impact of n-3 PUFA, the nutritional PPAR*α* ligands, on GSH metabolism. In WT mice, hepatic GSH concentration and pool are higher in mice fed the LIN diet than in those fed the COCO diet. This is consistent with previous studies suggesting that long-chain n-3 PUFA may exert a beneficial action on oxidative stress by increasing total glutathione in a rat model of chronic heart failure [26] and in cultured human fibroblasts [29]. However, to our knowledge, the present study is the first evidence that, in comparison with saturated fatty acids, even a very low amount of ALA may exert the same effects on GSH metabolism as its long-chain derivatives. We have shown previously that even a very low intake of dietary ALA (identical to the present study) activates typical targets of PPAR*α*, such as Cyp4a14 [9, 31]. However, the mechanisms by which ALA regulates GSH metabolism in WT mice remains speculative, since none of the genes studied, and in particular *γ*GCL, exhibited a difference in mRNA level between the COCO and the LIN diet. Thus, even if typical target genes of PPAR*α* are activated by the LIN diet, rich in 18 : 3 n-3, it is not possible to conclude on a direct involvement of PPAR*α* into the regulation of GSH level by fatty acids. Other pleiotropic effects of fatty acids have to be investigated, such as modifications of membrane microdomain composition (thus modulating receptors and ion channels functioning) or regulation of downstream cell signalling pathways. 

Unexpectedly, the impact of the dietary fatty acid profile on GSH metabolism was even more pronounced in KO mice. Indeed, *post-hoc* analysis showed that PPAR*α* deficiency increased total GSH content only in COCO-fed mice, and not in the LIN-fed mice. Specific activity and mRNA level of *γ*GCL were not affected by PPAR*α* deficiency in mice fed the COCO diet (Figure 1(a) and Table 4). Thus, their higher GSH content results not only from a difference in liver weight, which was 50% higher in this group than in the three other ones (Table 2), but also from their increased GSH concentration (Table 3), suggesting that the increased GSH pool in KO mice fed the COCO diet is diet specific. The mechanistic reasons why GSH concentration increased in KO mice when fed the COCO diet remain unclear, since *γ*GCL specific activity was the same as in their WT counterparts, whereas GPx activity was not affected by PPAR*α* deficiency. It may be hypothesised that, secondary to PPAR*α* invalidation, exportation of GSH into plasma towards extrahepatic tissues is impaired in KO mice, which would functionally affect the antioxidative defences of the whole body. In contrast, GSH concentration and pool were not significantly affected by PPAR*α* deficiency in mice fed the LIN diet (Table 3). To our knowledge, the only other study having investigated the consequence of PPAR*α* deficiency on GSH metabolism reported a significant depletion (20–25%) in total hepatic GSH content in fasted KO mice fed a standard rodent chow [23]. The fatty acid composition of the diet did not figure in this study, but was probably, as usual in standard rodent chows, soy oil rich in n-6 PUFA. Taken together, our data and the previous ones suggest that a diet in which the lipid moiety is rich in saturated FA and poor in PUFA, such as the COCO diet, makes the liver GSH pool sensitive to PPAR*α* invalidation, whereas PUFA would protect the mice against the effects of PPAR*α* deficiency.

**Figure 2:**
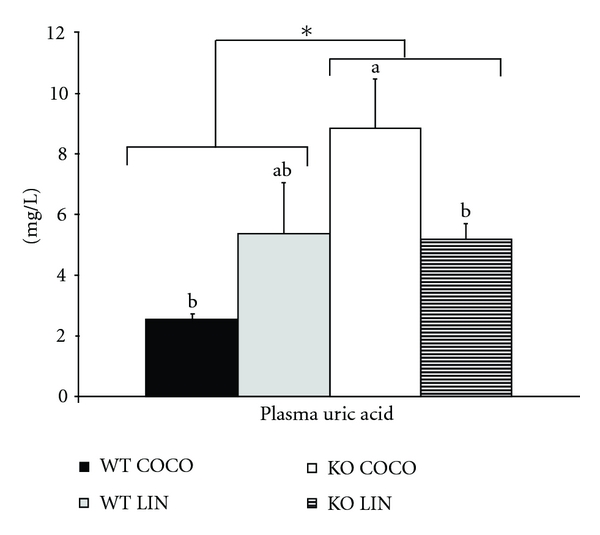
Plasma concentrations of uric acid in WT and PPAR*α*-deficient (KO) mice fed diets containing either saturated FA (COCO diet) or ALA (LIN diet) for 8 weeks. Values are means ± standard errors for 7 mice per group, *KO group significantly different from WT group *P* < 0.05. Columns sharing a same superscript letter, or without superscript letter, were not significantly different at *P* < 0.05.

In addition to the changes in cysteine metabolism and GSH status, PPAR*α* deficiency seemed to alter some markers of the oxidative and inflammatory status, in interaction with dietary fatty acids. Indeed, Sod2 (but not Sod1) mRNA level increased in KO mice, especially when fed the COCO diet (Table 4), which indicates an activation of antioxidant defences [44]. This is apparently inconsistent with the enhanced Sod2 expression by fenofibrate in mouse brain microvessels [45] and with the decrease in SOD activity in fasted PPAR*α* KO mice compared to the WT ones [23]. As discussed above for GSH content, these discrepancies may reflect an effect of the dietary fatty acid profile on the response to oxidative stress of PPAR*α* KO mice. In line with the higher Sod2 expression in PPAR*α* KO mice, we also observed an increase in the plasma concentration of uric acid, a widely recognized marker of oxidative stress [46], with the same diet ∗ genotype interaction: this concentration was 4-fold increased in KO mice fed the COCO diet, but not in those fed the LIN diet (Figure 2). To our knowledge, this is the first evidence of the impact of PPAR*α* deficiency of plasma uric acid concentration, in interaction with the dietary fatty acid profile. Altogether, these two markers (hepatic Sod2 mRNA level and plasma uric acid concentration) support the existence of a mild oxidative stress in PPAR KO mice, which is mitigated by the LIN diet. This is consistent with the previously reported prevention of hepatic steatosis in PPAR KO mice by ALA [30, 31]. Triglyceride accumulation in the liver is known to trigger oxidative stress, which in turn contributes to the pathogenesis of nonalcoholic steatohepatitis [47]. Therefore, a decrease in fatty acid accumulation in response to ALA feeding is expected to mitigate the oxidative stress resulting from PPAR*α* invalidation.

While PPAR deficiency induced a mild oxidative stress, evidences for inflammation were less conclusive, since the plasma concentration of both the proinflammatory cytokine MCP1 and the anti-inflammatory adipokine, adiponectine, was decreased, whereas PAI1 concentration did not change (Table 6). In the liver, mRNA levels of CD68, SAA, and MCP1 increased in KO mice, but the difference with WT mice was significant for CD68 only. Taken together, these results suggest a mild inflammatory status in response to PPAR*α* invalidation. This is consistent with previous results reporting that obesity-induced inflammation is aggravated in PPAR-deficient mice [48]. Finally, whatever the genotype, the plasma concentration of adiponectine, an anti-inflammatory adipokine, was higher in mice fed the LIN diet, suggesting an influence of the fatty acid profile of the diet, which is PPAR*α* independent.

## 5. Conclusion

Under our experimental conditions, and in accordance with a previous study [23], PPAR*α* deficiency seemed to induce an oxidative and inflammatory stress in the liver, as evidenced by the higher values of hepatic GSH pool and concentration, total *γ*GCL activity, Sod2 mRNA level, and plasma uric acid concentration. However, the phenotypic consequences of PPAR*α* deficiency depended, as last partly, on the dietary fatty acid profile. Indeed, most increases observed in KO mice fed the COCO diet (hepatic GSH pool and concentration, total *γ*GCL activity, Sod2 mRNA level, and plasma uric acid concentration) were alleviated, or even absent, in mice fed the LIN diet, suggesting that ALA would be protective against these effects of PPAR*α* invalidation. The reasons of this diet-based sensitivity remains unclear, but it is likely that it is not directly related to the PPAR*α* deficiency. More probably, it is secondary to the previously described effects of dietary fatty acids on hepatic steatosis in PPAR*α* KO mice [30, 31]. Indeed, this hepatic steatosis occurred in KO mice fed a SFA-containing diet, but was alleviated, and even absent, in those fed a PUFA-containing diet. It may thus be hypothesized that the beneficial effects of dietary PUFA on liver metabolism in KO mice, even in low amounts, may be accounted for by a protection against lipid accumulation, resulting in lower lipotoxicity and oxidative stress than with SFA.

## Figures and Tables

**Table 1:** Primer sequences used in quantitative RT-PCR analysis.

Gene name	Abbreviation	Ref Seq	Forward primer	Reverse primer
Glutathione peroxidase 1 (GPX1)	Gpx1	NM_008160	GACACCAGGAGAATGGCAAGA	ACCATTCACTTCGCACTTCTCA
Cysteine dioxygenase (CDO)	Cdo	NM_033037	GATACATGCCACGCCTTTGA	CCTGAAGTTGTAAATGGAGTCCTGAT
Catalase (CAT)	Cat	NM_009804	GCCAGAAGAGAAACCCACAGACT	CACTGAACAAGAAAGAAACCTGATG
Glutamate cysteine ligase (*γ*GCL), catalytic subunit	Gclc	NM_010295	GGAGGCGATGTTCTTGAGACTCT	CCTTCGATCATGTAACTCCCATACT
Glutamate-cysteine ligase (*γ*GCL), modifier subunit	Gclm	NM_008129	GGCCTCCTGCTGTGTGATG	GCCTCAGAGAGCAGTTCTTTCG
Superoxide dismutase 1 (SOD1)	Sod1	NM_011434.1	GTGCAGGGAACCATCCACTT	GTCCTGACAACACAACTGGTTCA
Superoxide dismutase 2 (SOD2)	Sod2	NM_013671	GCTCTGGCCAAGGGAGATG	TGATTAATATGTCCCCCACCATT
CD68 antigen (CD68)	Cd68	NM_009853.1	CATCAGAGCCCGAGTACAGTCTACC	AATTCTGCGCCATGAATGTCC
Chemokine (C-C motif) ligand 2 (MCP1)	Ccl2	NM_011333.3	GGCTCAGCCAGATGCAGTTAA	CCAGCCTACTCATTGGGATCA
Serum amyloid A (SAA)	Saa	NM_009117.3	GCGAGCCTACACTGACATGA	TTTTCTCAGCAGCCCAGACT

All primer sequences were designed using Primer Express (Applied Biosystems) software and were from Eurogentec (Eurogentec, Seraing, Belgium).

**Table 2:** Markers of PPAR*α* deficiency in WT and PPAR*α*-null (KO) mice fed diets containing either saturated FA (COCO diet) or ALA (LIN diet) for 8 weeks.

	WT		KO		*P* values			
	COCO	LIN	COCO	LIN	ANOVA	Genotype (G)	Diet (D)	Interaction G∗D
Body weight (g)	29.5 ± 0.5^b^	29.6 ± 0.5^b^	36.7 ± 1.0^a^	32.7 ± 1.5^b^	0.0001	0.0001	0.0379	0.0261
Liver weight (g)	1.07 ± 0.04^b^	1.07 ± 0.06^b^	1.55 ± 0.08^a^	1.04 ± 0.04^b^	<0.0001	0.0004	0.0001	0.0001
Liver weight (%)	3.88 ± 0.10^b^	3.88 ± 0.25^b^	4.50 ± 0.12^a^	3.71 ± 0.12^b^	0.0007	0.1403	0.0112	0.0123
EpAT weight (%)	2.31 ± 0.16^b^	2.58 ± 0.19^ab^	3.47 ± 0.28^a^	2.91 ± 0.40^ab^	0.0013	0.0038	0.5062	0.0720
IngAT weight (%)	1.46 ± 0.19^b^	1.78 ± 0.12^ab^	2.50 ± 0.23^a^	1.79 ± 0.22^ab^	0.0063	0.0090	0.3293	0.0102
Blood glucose (g/L)	1.94 ± 0.18^b^	1.83 ± 0.21^b^	1.48 ± 0.17^ab^	1.17 ± 0.12^a^	0.0001	0.0001	0.1892	0.4690
Plasma TG (mg/dL)	0.25 ± 0.02^a^	0.30 ± 0.03^ab^	0.39 ± 0.01^b^	0.36 ± 0.05^ab^	0.0231	0.0039	0.7881	0.3064
Plasma CT (mgd/L)	0.75 ± 0.02^b^	0.62 ± 0.05^b^	1.20 ± 0.07^a^	0.84 ± 0.11^b^	0.0001	0.0001	0.0015	0.0823

Values are means ± standard errors for 7 mice per group. Mean values within a row sharing a same superscript letter, or without superscript letter, were not significantly different at *P* < 0.05.

**Table 3:** Hepatic thiols concentrations and pools in WT and PPAR*α*-null (KO) mice fed diets containing either saturated FA (COCO diet) or ALA (LIN diet) for 8 weeks.

	WT		KO		*P* values			
	COCO	LIN	COCO	LIN	ANOVA	Genotype (G)	Diet (D)	Interaction G∗D
GSH (*μ*mol/g of protein)	3 112 ± 180^b^	5 138 ± 412^a^	5 201 ± 509^a^	3 892 ± 382^ab^	0.0013	0.2815	0.3569	0.0003
Cysteine (*μ*mol/g of protein)	274 ± 91.8	190 ± 66.0	211 ± 55.8	243 ± 55.8	0.8377	0.9432	0.7061	0.4144
CysGly (*μ*mol/g of protein)	97.5 ± 5.80	83.6 ± 12.7	102 ± 20.5	93.5 ± 8.14	0.8012	0.6243	0.4461	0.8514
GSH (*μ*mol/ liver)	439 ± 31.3^b^	774 ± 77.2^ac^	1031 ± 118^a^	544 ± 80.7^bc^	0.0002	0.0367	0.3590	<.0001
Cysteine (*μ*mol/ liver)	40.2 ± 16.0	29.3 ± 10.6	41.3 ± 10.1	33.2 ± 7.62	0.8614	0.8304	0.4181	0.9034
CysGly (*μ*mol/ liver)	14.2 ± 1.24	13.1 ± 2.03	20.0 ± 3.49	12.9 ± 1.19	0.1456	0.2600	0.1099	0.2383

Values are means ± standard errors for 7 mice per group. Mean values within a row sharing a same superscript letter, or without superscript letter, were not significantly different at *P* < 0.05.

**Table 4:** Hepatic mRNA levels of cysteine and glutathione metabolism key genes, and of inflammatory markers, in WT and PPAR*α*-null (KO) mice fed diets containing either saturated FA (COCO diet) or ALA (LIN diet) for 8 weeks (arbitrary units).

	WT		KO			*P* values		
	COCO	LIN	COCO	LIN	ANOVA	Genotype (G)	Diet (D)	Interaction G∗D
Glutamate cysteine ligase (*γ*GCLc), catalytic subunit	0.22 ± 0.04	0.30 ± 0.07	0.22 ± 0.05	0.13 ± 0.02	0.1583	0.0941	0.9836	0.1183
Glutamate cysteine ligase (*γ*GCLm), modifier subunit	0.68 ± 0.11	0.65 ± 0.07	0.55 ± 0.07	0.63 ± 0.17	0.8840	0.5255	0.8070	0.6317
Cysteine dioxygenase (CDO)	3.84 ± 0.61	2.24 ± 0.16	2.22 ± 0.38	3.92 ± 1.02	0.1193	0.8885	0.9780	0.0181
Glutathione peroxidase 1 (GPX1)	8.65 ± 1.58	9.32 ± 1.15	8.79 ± 1.55	9.54 ± 2.71	0.9857	0.9272	0.7211	0.9844
Catalase (CAT)	1.05 ± 0.28	0.97 ± 0.12	0.92 ± 0.22	0.71 ± 0.13	0.6463	0.3327	0.4750	0.7445
Superoxide dismutase 1 (SOD1)	64.9 ± 3.36	61.2 ± 3.68	63.1 ± 3.95	69.8 ± 3.96	0.4529	0.3941	0.7101	0.2006
Superoxide dismutase 2 (SOD2)	5.09 ± 1.06^b^	5.66 ± 0.77^bc^	10.1 ± 0.63^a^	8.49 ± 0.7^ac^	0.0005	<0.001	0.517	0.1873
CD68 antigen (CD68)	20.0 ± 10.4^ab^	8.75 ± 8.28^b^	30.9 ± 11.9^a^	29.1 ± 14.8^a^	0.0121	0.0027	0.1717	0.3142
Chemokine (C-C motif) ligand 2 (MCP1)	0.94 ± 0.45	0.77 ± 0.25	1.45 ± 0.28	1.55 ± 1.77	0.3467	0.0898	0.7011	0.9267
Serum amyloid A (SAA)	0.47 ± 0.29	0.51 ± 0.36	2.13 ± 3.83	2.70 ± 2.69	0.2919	0.0684	0.7926	0.7615

Gene expression was determined using the 2^−ΔCt^ formula where ΔCt = (Ct  target  gene − Ct  18S). Values are means ± standard errors for 4–7 mice per group. Mean values within a row sharing a same superscript letter, or without superscript letter, were not significantly different at *P* < 0.05.

**Table 5:** Plasma concentrations of amino acid involved in cysteine metabolism in WT and PPAR*α*-null (KO) mice fed diets containing either saturated FA (COCO diet) or ALA (LIN diet) for 8 weeks.

	WT		KO		*P* values			
	COCO	LIN	COCO	LIN	ANOVA	Genotype (G)	Diet (D)	Interaction G∗D
Cysteine	16.3 ± 3.64	15.0 ± 2.12	17.2 ± 2.71	17.6 ± 3.87	0.9574	0.6144	0.8946	0.7949
Glycine	231 ± 17.3^a^	219 ± 14.26^ab^	170 ± 5.85^b^	214 ± 16.3^ab^	0.0236	0.0250	0.2603	0.0600
L-glutamic acid	23.6 ± 1.20	23.9 ± 3.09	21.5 ± 1.38	19.4 ± 1.20	0.3186	0.0800	0.6746	0.5300
Methionine	39.8 ± 1.89	38.9 ± 2.07	47.1 ± 3.83	49.8 ± 5.32	0.0980	0.0150	0.8094	0.6253
Taurine	436 ± 59.8	511 ± 38.0	389 ± 43.0	366 ± 22.4	0.1290	0.0403	0.5624	0.2797

Values are expressed in *μ*M. Values are means ± standard errors for 7 mice per group. Mean values within a row sharing a same superscript letter, or without superscript letter, were not significantly different at *P* < 0.05.

**Table 6:** Plasma hormones and cytokines concentrations in WT and PPAR*α*-null (KO) mice fed diets containing either saturated FA (COCO diet) or ALA (LIN diet) for 8 weeks.

	WT		KO		*P* values			
	COCO	LIN	COCO	LIN	ANOVA	Genotype (G)	Diet (D)	Interaction G∗D
Insulin (ng/mL)	1.68 ± 0.36	2.87 ± 0.54	2.66 ± 0.37	1.83 ± 0.77	0.2300	0.9510	0.7145	0.0531
Leptin (pg/mL)	222 ± 52.9	254 ± 71.54	179 ± 39.2	242 ± 57.9	0.7748	0.6541	0.4430	0.8041
Adiponectine (mg/mL)	8.11 ± 1.03^b^	13.6 ± 1.37^a^	6.95 ± 0.45^b^	10.7 ± 0.81^ab^	0.0005	0.0596	0.0002	0.3990
MCP 1 (pg/mL)	20.2 ± 4.55	18.3 ± 1.75	10.3 ± 1.89	13.8 ± 1.69	0.1040	0.0338	0.8119	0.4066
PAI 1 (ng/mL)	1.53 ± 0.19	1.65 ± 0.31	0.97 ± 0.21	1.65 ± 0.43	0.2241	0.3203	0.1616	0.3120

Values are means ± standard errors for 7 mice per group. Mean values within a row sharing a same superscript letter, or without superscript letter, were not significantly different at *P* < 0.05.
